# Language and Race

**Published:** 1894-11-10

**Authors:** 


### The Study of Man.

### VIII.—LANGUAGE AND RACE.

Probably no one has crossed from Dover to Ostend, or from Queensborough to Flushing, without being agreeably surprised to hear on his arrival, not, as at Calais or Boulogne, a babel of strange words and stranger "accent," but a dialect which, at first hearing, sounds not unlike the peasant-English of the eastern counties, and in which he can recognise, after a few hours' practice, a number of words and grammatical forms which hardly differ from those of his native tongue. And when, at the same time, he recognises in the Dutch or Flemish streets, familiar types of figure, feature, and complexion, he will inevitably, and rightly, conclude that a people who speak a language so like his own cannot be wholly unconnected in race, too, with his own countrymen.

If he passes, further, from the Low Countries into Germany, or through Denmark into Scandinavia, he will again be prepared, on the ground of similar likenesses of speech to recognise similar blood-relationships between Dutch, Flemings, and Germans, and again between these and Danes and Scandinavians; and to rank all the leading peoples of the north-west of Europe in one "Teutonic" family. Quite as obvious are the connections respectively between the "Slavonic" dialects of the north-east and between the languages of France, Italy, and Spain; and here, too, in greater or less degree, the physical characteristics of the peoples bear out the conclusion which suggests itself.

But let our traveller pass rapidly from the south of Italy or the south of Spain, through Switzerland, or the Riviera, or the borders of the Basque country, into the centre and west and north of France, and he will certainly note from time to time changes in the physiognomy of the people which do not correspond, and often conflict, with the evidence of the dialect which they speak. Now let him go to Scotland or Ireland, to Wales, to Cornwall, or to Brittany, and hear men of strongly-marked "Celtic" builds speaking "Teutonic" English; better still, let him go to America, and hear African negroes or Brazilian aborigines speaking English or Spanish as their mother tongues, and knowing no other. Is it still quite so clear that community of language means community of race?

It has been only within this century that the questions here indicated have been raised in an answerable form; and that the study of language has joined forces with the study of man for their solution. It is true that, as early as 1477, a studious German collected, for purposes of linguistic comparison, the known translations of the Lord's Prayer; of which a far larger collection will be found in the "Mithridates" of J. C. Adelung (1806-16), the first systematic attempt to interpret the facts of language by the comparison of parallel passages, and not only through the likeness of individual words, such as are collected in the "St. Petersburg Dictionary" of the Empress Catherine, and in the vocabulary projected even earlier by the philosopher Leibnitz.

But little could be done until the extension of trade and colonisation had made the study of foreign languages both easier and of more importance; until the classical scholars of Europe had both the inducement and the will to join in the interpretation of them; and, in particular, until the publication of Sanskrit literature, by Sir William Jones (1786) and other Orientalists, revealed in the far East a language quite as closely related to Latin and Greek as these are to one another. The affinities between languages, however, once thus exemplified, became at once the field for a new range of research and speculation, the fruits of which are in many departments even yet not fully ripe.

In 1808 F. W. Schlegel, one of the first to appreciate the importance of Sanskrit in philology, constructed and published a genealogical arrangement of a large number of races, on the assumption that Sanskrit stood in the relation of a "mother-tongue" to Latin, Greek, and the allied languages of Europe; while a similar position was claimed for Old Persian (Zend) by H. F. Link in 1821. It soon became evident, however, that these ancient languages stood to one another rather as sister-tongues; and, together with Greek, Latin, and its derivative Romance Languages, and the Slavonic, Teutonic, and Celtic groups, formed, as it were, one family, to be derived, if at all, from a lost ancestral "mother-tongue," which might exhibit the fundamental characteristics of each of them. The family of languages thus constituted has been variously termed Aryan or Indo-Germanic, and has by the majority of philologists been assumed to correspond to an Indo-Germanic group of allied races. It remains to be seen how far the inner arrangement of the linguistic group can be trustworthily established, and how far the conception of a migratory Aryan race connects and explains the data of anthropology.

The method, however, when once initiated, was soon applied to other groups of languages; and Crawfurd's "History of the Indian Archipelago," published as early as 1820, contains an essay on the genealogical classification of the Polynesian dialects.

The Semitic group also, of which Hebrew and Arabic are the best-known representatives, affords special facilities for this kind of study.

Other groups, such as the languages of the Turks and Tartars, and of the Finns and other tribes of Northern Asia, have hardly yet been sufficiently analised to admit of such inferences to ethnic relationship. In both the cases quoted all that is clear is that the peoples in question had originally but a very poor vocabulary, and enriched themselves, in both the material and the nomenclature of civilisation, by loans from their more cultivated neighbours. On the other hand, in the group to which the Chinese belong, it is the structure rather than the vocabulary of the language which gives the clearest indication of development and historical relationship.

Now, what are the leading principles of the method which thus interprets the results of the study of language into a direct contribution to the study of man? This will probably be made most clear by setting out at length a single instance; and it happens that the best instance is also that which affects our own history most nearly. But the moral of the "Aryan Controversy" is not to be compressed into a single paragraph.

